# *PNPLA3*-Ile148Met and *TM6SF2*-Glu167Lys increase susceptibility to metabolic dysfunction-associated steatotic liver disease in children

**DOI:** 10.3389/fendo.2025.1689656

**Published:** 2025-10-31

**Authors:** Hengpan Yao, Zhiyi Xia, Yijing Liu, MengJun Dong, Kairui Yang, Fang Zhou

**Affiliations:** ^1^ Department of Digestion, Children’s Hospital Affiliated to Zhengzhou University, Henan Children’s Hospital Zhengzhou Children’s Hospital, Zhengzhou, Henan, China; ^2^ Henan Provincial Key Laboratory of Children's Genetics and Metabolic Diseases, Henan Children’s Hospital, Zhengzhou, Henan, China

**Keywords:** MASLD, PNPLA3, TM6SF2, pediatric hepatology, genetics, polymorphism, genetic susceptibility

## Abstract

**Background:**

With the rising global prevalence of childhood obesity, the incidence of metabolic dysfunction-associated steatotic liver disease (MASLD) in pediatric and adolescent populations is also increasing. The genetic mechanisms underlying MASLD remain incompletely elucidated. This study aimed to investigate the roles of eight genetic loci—*PNPLA3*-Ile148Met, *PNPLA3*-Lys434Glu, *GCKR*-Leu446Pro, *TM6SF2*-Glu167Lys, *LEPR*-Lys109Arg, *LEPR*-Lys656Asn, *IRS1*-Gly971Arg, and *KLB*-Arg728Gln—in the susceptibility to MASLD in Chinese children and adolescents, in order to provide scientific evidence for genetic research on MASLD. We hypothesized that *PNPLA3*-Ile148Met and *TM6SF2*-Glu167Lys variants confer susceptibility to MASLD in children.

**Methods:**

A total of 350 children and adolescents aged 7–17 years were enrolled, including 196 with MASLD (case group) and 154 healthy controls. Demographics, medical history, anthropometric measurements, and hepatic B-ultrasound data were collected. Fasting morning blood samples were obtained for biochemical analysis and genotyping. Statistical analyses included chi-square tests, Fisher’s exact tests, and multivariate logistic regression to identify predictive factors for pediatric MASLD.

**Results:**

The allele frequencies of *PNPLA3*-Ile148Met, *PNPLA3*-Lys434Glu, and *TM6SF2*-Glu167Lys were significantly higher in the MASLD group than in the control group (*PNPLA3*-Ile148Met and *TM6SF2*-Glu167Lys: both P_FDR < 0.001; *PNPLA3*-Lys434Glu: P_FDR=0.024), indicating an association with increased MASLD risk. In contrast, the allele frequencies of *GCKR*-Leu446Pro and *LEPR*-Lys109Arg were significantly lower in the MASLD group (P_FDR=0.009), suggesting potential protective effects. Multivariate logistic regression identified male sex, the *PNPLA3*-Ile148Met-GG genotype, and the *TM6SF2*-Glu167Lys-CT genotype as independent risk factors for MASLD. Additionally, carriers of the *PNPLA3* rs738409 GG genotype exhibited significantly higher levels of AST, ALT, and TC compared to those with the GC genotype (all P_FDR < 0.001).

**Conclusion:**

The *PNPLA3*-Ile148Met and *TM6SF2*-Glu167Lys gene polymorphisms are independent risk factors that significantly increase the risk of MASLD in Chinese children. Additionally, the *PNPLA3*-Lys434Glu variant is associated with increased risk at the allele level. In contrast, *GCKR*-Leu446Pro and *LEPR*-Lys109Arg may confer protective effects. This study provides new evidence for genetic susceptibility to MASLD in the pediatric population.

## 1 Introduction

The nomenclature of fatty liver disease has evolved through three phases: from “non-alcoholic fatty liver disease” (NAFLD) to “metabolically associated fatty liver disease” (MAFLD), and finally to the current standardized term MASLD. To better reflect its pathophysiological mechanisms, international consensus recommends renaming the former “NAFLD” to “MASLD”, removing the terms “non-alcoholic” and “fatty” to align with specific diagnostic criteria. For the convenience of future academic research and communication, this study adopts the revised MASLD designation (1–3). Pediatric MASLD is an important public health issue worldwide (4, 5). MASLD has become the most prevalent chronic liver disorder among children and adolescents worldwide (6), with its prevalence closely mirroring the obesity epidemic. Studies indicate a global prevalence rate of 13% in children and adolescents, while rates soar to 47.00% in a special population based on child obesity respectively (7). This health crisis is no longer confined to developed nations, as its prevalence in low-and middle-income countries is rising at an unprecedented rate. These patterns are directly linked to the global dietary shift toward Westernized diets (high-sugar, high-fructose beverages, and processed foods) and the widespread adoption of sedentary lifestyles. MASLD is increasingly being seen in children in China. Related analysis revealed that the total prevalence of MASLD in Chinese children is 6.30%, and the prevalence of overweight and obese children is 40.4%. MASLD is a clinicopathological syndrome characterized by hepatic steatosis, excluding ethanol and other definite liver damage factors (8). Recent studies have shown that the development of MASLD is associated with lipid accumulation, oxidative stress, gut microbiota imbalance and genetic factors (9, 10). Amid evolving lifestyles, dietary structures, and escalating obesity in younger populations, MASLD has emerged as the leading chronic liver disorder. The importance of MASLD in children goes far beyond the liver itself. It is a disease that affects multiple systems. Apart from liver-related morbidity, MASLD further associates with heightened incidence of cardiovascular disease, type 2 diabetes, and fatal outcomes (11). Genetic factors critically contribute to MASLD pathogenesis, given that not every obese child develops the condition (12). *PNPLA3*-Ile148Met (rs738409, patatin-like phospholipase domain-containing protein 3) is the most impactful genetic risk factor for steatotic liver disease (13, 14). Moreover, some meta-analyses have suggested that this variant is significantly associated with elevated serum alanine transaminase, aspartate transaminase, and gamma-glutamyltransferase concentrations and liver fat content (15, 16). *TM6SF2* is involved in triglyceride secretion and the regulation of hepatic lipid metabolism (17, 18). When a mutation occurs at the *TM6SF2*-Glu167Lys site, it increases the risk of hepatic fat accumulation and liver injury.

However, despite growing evidence on genetic susceptibility to MASLD, most studies have focused on adult or non-East Asian populations, leaving a significant knowledge gap regarding genetic risk factors in Chinese children. To address this, we systematically selected eight single nucleotide polymorphisms (SNPs)—previously associated with MASLD in other populations but underexplored in Chinese pediatric cohorts—with minor allele frequency >0.10, aiming to identify susceptibility loci specific to this demographic. Our results demonstrate that *PNPLA3*-Ile148Met, *PNPLA3*-Lys434Glu, and *TM6SF2*-Glu167Lys significantly increase MASLD risk in Chinese children, thereby filling a critical research gap in this population. Notably, while *GCKR*-Leu446Pro has been widely reported as a risk locus in Western pediatric studies, it exhibited a protective effect in our cohort, highlighting the importance of population-specific genetic investigations.

## 2 Materials and methods

### 2.1 Data acquisition

A total of 350 children were enrolled in this study, including 196 children and adolescents with MASLD as the experimental group and 154 healthy children and adolescents as the control group. All participants and their legal guardians provided written informed consent. The study was approved by the Medical Ethics Committee of the Children’s Hospital Affiliated to Zhengzhou University, Henan Province(Approval No.: 2024-071-002). All participants were unrelated and of Han Chinese ethnicity.

### 2.2 Selection of 8 polymorphic loci

The 8 target SNPs were selected based on a comprehensive consideration of three key factors: (1) Sample size adaptability: Given the total sample size of 350 (196 in the experimental group and 154 in the control group), loci with a minor allele frequency > 0.10 were prioritized to avoid insufficient statistical power caused by rare variants; (2) Population frequency relevance: Loci with well-documented frequency data in Chinese populations (especially pediatric subgroups) were selected to ensure consistency with the study’s ethnic background; (3) MASLD association evidence: Only loci previously reported to be associated with MASLD or its pathological mechanisms (e.g., hepatic lipid metabolism, steatosis, and metabolic dysfunction) in international or Chinese adult studies were included, to focus on genetically meaningful targets for pediatric MASLD validation.

### 2.3 Sequencing and genotyping

Peripheral blood samples (200 μL) were collected from all participants, and genomic DNA was extracted using a commercial DNA extraction kit (Sangon Biotech, Cat. No.: B518253, Shanghai, China) following the manufacturer’s instructions; multiplex amplification primers were then designed based on the genomic positions of all 8 target SNPs to achieve targeted capture of the SNP regions. High-throughput sequencing was performed on the Illumina HiSeq X platform to generate 150 bp paired-end reads; raw sequencing data were filtered for quality (e.g., removing low-quality reads with Phred scores < 20), and the remaining high-quality reads were aligned to the human reference genome (GRCh37—hg19) using the bwa-mem2-v2.2.1 algorithm (19). Genotypes were finally identified using BCFtools-v1.9 (via the mpileup function) to generate genomic Variant Call Format (gVCF) files, which included both variant and wild-type genotype information for all target SNPs (20).

### 2.4 Liver ultrasound examination and MASLD diagnosis

#### 2.4.1 Diagnostic criteria

MASLD was diagnosed per the Expert Consensus on the Diagnosis and Management of Metabolic Dysfunction-Associated Steatotic Liver Disease in Children (2025): (1) Ultrasonographic evidence of hepatic steatosis (e.g., liver-kidney echo discrepancy, attenuated echo penetration); (2) At least one cardiovascular/metabolic risk factor (e.g., BMI ≥ 85th percentile, abnormal blood glucose/lipids); (3) Exclusion of other liver injury causes (e.g., inherited metabolic disorders, drug-induced liver injury) (1).

#### 2.4.2 Rationale for liver ultrasound

Liver ultrasound was used as it is non-invasive, safe for children, cost-effective, and widely accessible—consistent with international guidelines recommending it as the first-line tool for pediatric MASLD screening to identify clinically significant steatosis.

### 2.5 Statistical analysis and graphing

Statistical analyses were conducted using SPSS-v21.0, and graphs were generated via Python’s Matplotlib (v3.5). Hardy–Weinberg equilibrium (HWE) tests were performed for each SNP: Pearson χ² test was used if expected genotype counts ≥ 5, and Fisher’s two-tailed exact test was used if < 5. SNP allele/genotype frequency differences between groups were analyzed via chi-square test or Fisher’s exact test, as appropriate.

### 2.6 Genetic model selection for multivariate analysis

To ensure a robust and pre-specified approach for identifying independent genetic risk factors, we determined the most appropriate genetic model for each SNP prior to multivariate analysis. For all eight SNPs, we fitted univariate logistic regression models under additive, dominant, and recessive genetic models. The optimal model for each SNP was selected based on the lowest Akaike Information Criterion (AIC), indicating the best fit to the data. This pre-analysis led to the following model specifications for the final multivariate model:

1. *PNPLA3*-Ile148Met: A recessive model was used. This was represented by a dummy variable comparing the homozygous risk genotype (GG) against the combined group of heterozygotes and wild-type homozygotes (GC + CC).
2. *TM6SF2*-Glu167Lys: A dominant model was used. This was represented by a dummy variable comparing carriers of at least one risk allele (CT + TT) against wild-type homozygotes (CC).

Multivariable logistic regression (with MASLD status as the dependent variable) was then employed to identify independent predictive factors, incorporating the pre-specified genetic models for *PNPLA3*-Ile148Met and *TM6SF2*-Glu167Lys, alongside covariates including age, sex, and BMI. Variable selection was performed using a stepwise method.

**Figure 1:**
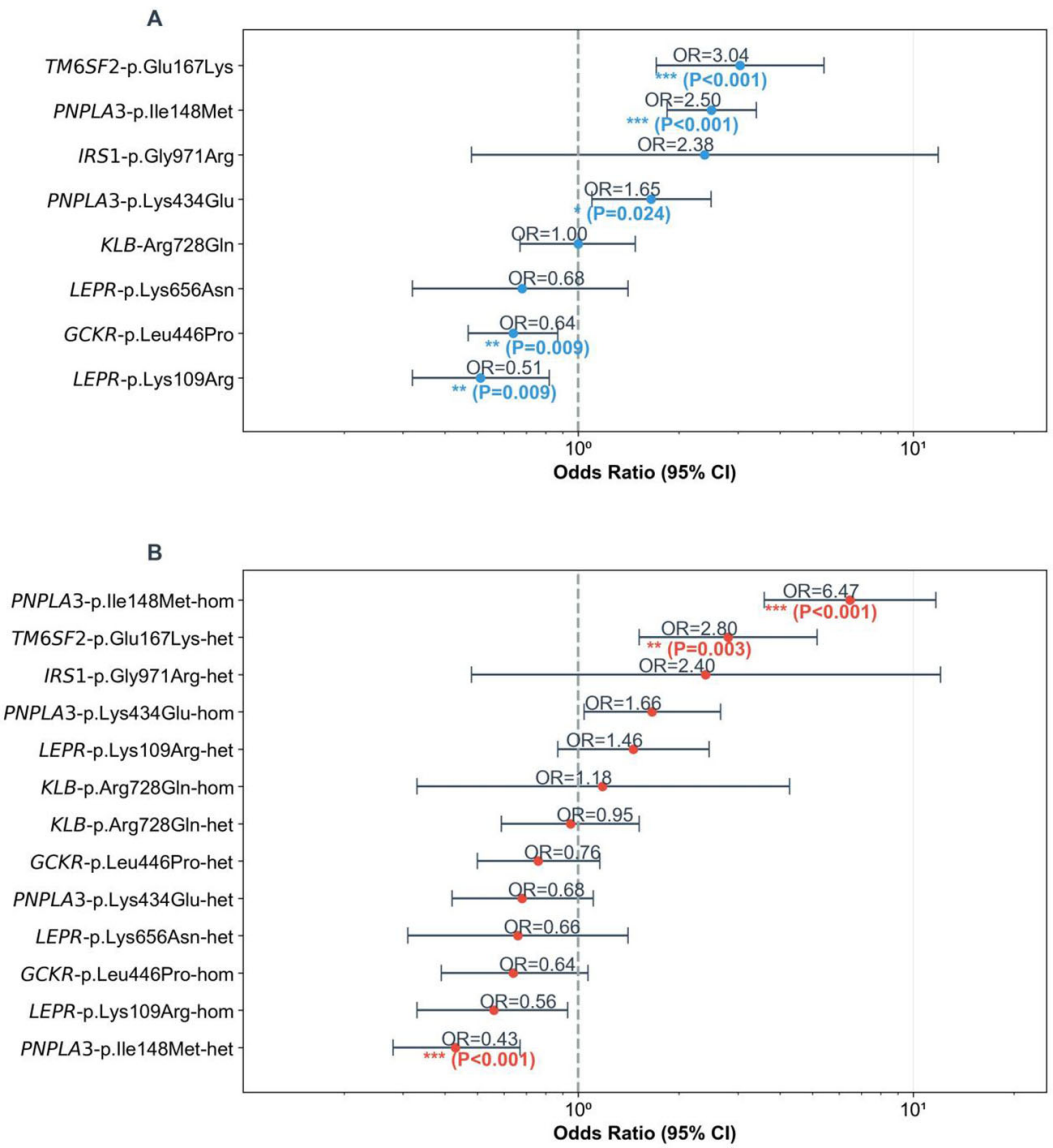
Forest plots of SNP associations with childhood MASLD. **(A)** ORs (MASLD/control) with 95% CIs for allele frequencies of 8 SNPs and MASLD. Dashed line = OR = 1; asterisks mark significance (***P<0.001, **P<0.010, *P<0.050). **(B)** Odds ratios (ORs, MASLD/control) with 95% CIs for homozygous/heterozygous genotypes of 8 SNPs and MASLD. Dashed line = OR = 1 (no association); asterisks mark significance. (***P<0.001, **P<0.010, *P<0.050).

Differences in biochemical indicators between *PNPLA3*-p.Ile148Met CG/GG genotypes were analyzed via Mann–Whitney U test (due to non-normal distribution). To control for type I errors in multiple testing, we applied false discovery rate (FDR) correction using the Benjamini–Hochberg procedure with the following specific implementations:

1. For genetic association analyses (**Tables 1**, **2**, **Figure 1**): We performed separate FDR corrections for: The 8 SNP allele frequency comparisons (**Table 1**); The 13 genotype frequency comparisons (**Table 2**); Each set was corrected independently at α = 0.05 using the multipletests function in Python’s statsmodels library (method=‘fdr_bh’).
2. For biochemical marker comparisons (**Table 3**): Given the examination of 16 different biochemical parameters between genotype groups, we applied both FDR correction (primary method) and Bonferroni correction (secondary, more conservative method). The FDR threshold was set at q < 0.05.
3. For multivariate logistic regression (**Table 4**): P-values for the 5 predictor variables in the final model were subjected to FDR correction.

**Table 1:** Allele frequency distribution and association analysis results of 8 SNPs between MASLD and control groups in children.

Allele	MASLD VAF (%)	Ctrl VAF (%)	P_Raw	P_FDR	OR(95%CI)
*PNPLA3*-p.Ile148Met;C-G	58.16 (228/392)	35.71 (110/308)	<0.001	<0.001	2.50 (1.84-3.40)
*PNPLA3*-p.Lys434Glu;A-G	87.24 (342/392)	80.52 (248/308)	0.015	0.024	1.65 (1.10-2.49)
*GCKR*-p.Leu446Pro;T-C	40.31 (158/392)	51.30 (158/308)	0.004	0.009	0.64 (0.47-0.87)
*TM6SF2*-p.Glu167Lys;C-T	14.29 (56/392)	5.19 (16/308)	<0.001	<0.001	3.04 (1.71-5.42)
*LEPR*-p.Lys109Arg;A-G	83.67 (328/392)	90.91 (280/308)	0.005	0.009	0.51 (0.32-0.82)
*LEPR*-p.Lys656Asn;G-C	3.57 (14/392)	5.19 (16/308)	0.292	0.390	0.68 (0.32-1.41)
*IRS1*-p.Gly971Arg;C-T	1.53 (6/392)	0.65 (2/308)	0.477	0.544	2.38 (0.48-11.87)
*KLB*-p.Arg728Gln;G-A	16.84 (66/392)	16.88 (52/308)	0.987	0.987	1.00 (0.67-1.48)

**Table 2:** Genotype frequency differences of SNPs between MASLD and control groups in children.

Genotype	MASLD_freq(%)	Ctrl_freq(%)	P_Raw	P_FDR	OR(95%CI)
*PNPLA3*-p.Ile148Met;CG	30.61(60/196)	50.65(78/154)	<0.001	<0.001	0.43(0.28-0.67)
*PNPLA3*-p.Ile148Met;GG	42.86(84/196)	10.39(16/154)	<0.001	<0.001	6.47(3.59-11.67)
*PNPLA3*-p.Lys434Glu;AG	21.43(42/196)	28.57(44/154)	0.123	0.234	0.68(0.42-1.11)
*PNPLA3*-p.Lys434Glu;GG	76.53(150/196)	66.23(102/154)	0.033	0.093	1.66(1.04-2.66)
*GCKR*-p.Leu446Pro;TC	43.88(86/196)	50.65(78/154)	0.208	0.291	0.76(0.50-1.16)
*GCKR*-p.Leu446Pro;CC	18.37(36/196)	25.97(40/154)	0.087	0.202	0.64(0.39-1.07)
*TM6SF2*-p.Glu167Lys;CT	24.49(48/196)	10.39(16/154)	<0.001	0.003	2.80(1.52-5.16)
*LEPR*-p.Lys109Arg;AG	24.49(48/196)	18.18(28/154)	0.155	0.242	1.46(0.87-2.46)
*LEPR*-p.Lys109Arg;GG	71.43(140/196)	81.82(126/154)	0.024	0.084	0.56(0.33-0.93)
*LEPR*-p.Lys656Asn;GC	7.14(14/196)	10.39(16/154)	0.281	0.358	0.66(0.31-1.41)
*IRS1*-p.Gly971Arg;CT	3.06(6/196)	1.30(2/154)	0.474	0.553	2.40(0.48-12.06)
*KLB*-p.Arg728Gln;GA	27.55(54/196)	28.57(44/154)	0.833	0.897	0.95(0.59-1.52)
*KLB*-p.Arg728Gln;AA	3.06(6/196)	2.60(4/154)	>0.999	>0.999	1.18(0.33-4.27)

**Table 3:** Differential analysis of age, BMI and blood-related indicators between *PNPLA3* - p.Ile148Met;CG and GG Genotype Groups.

Variable	*PNPLA3*-p.Ile148Met;CG Median (IQR) (n=60)	*PNPLA3*-p.Ile148Met;GG Median (IQR) (n=84)	P_Raw	P_FDR
Age	11.56(10.06-13.18)	11.16(10.21-12.53)	0.791	0.847
BMI	27.01(25.25-28.57)	27.85(24.25-31.15)	0.550	0.733
AST(U/L)	43.84(25.09-63.29)	62.76(37.94-105.84)	<0.001	<0.001
ALT(U/L)	56.26(35.87-109.01)	118.35(71.31-204.92)	<0.001	<0.001
GGT(U/L)	32.37(22.93-48.01)	29.89(18.75-50.87)	0.847	0.847
Glucose(mmol/L)	5.12(4.84-5.44)	5.16(4.84-5.38)	0.822	0.847
Insulin(uU/mL)	20.72(11.89-28.41)	21.58(12.03-31.49)	0.159	0.459
TG(mmol/L)	1.26(1.00-1.56)	1.12(0.78-1.86)	0.201	0.459
TBIL(umol/L)	8.69(6.88-10.38)	9.49(7.05-12.18)	0.176	0.459
DBIL(umol/L)	2.08(1.38-2.71)	2.02(1.04-3.02)	0.293	0.547
IBIL(umol/L)	8.70(6.93-10.68)	8.06(5.80-9.89)	0.344	0.550
HDL(mmol/L)	1.34(1.08-1.56)	1.30(0.98-1.54)	0.847	0.847
LDL(mmol/L)	2.06(1.54-2.39)	2.30(1.82-2.94)	0.080	0.321
TC(mmol/L)	3.68(3.28-4.08)	4.21(3.62-4.89)	<0.001	<0.001
ApoA(g/L)	1.39(1.28-1.52)	1.34(1.18-1.53)	0.502	0.730
ApoB(g/L)	0.73(0.66-0.84)	0.81(0.70-0.93)	0.308	0.547

Data are presented as median (25th, 75th) or percent. BMI, body mass index; ALT, alanine aminotransferase; AST,  aspartate aminotransferase; GGT, γ-glutamyltransferase; TG, triglyceride; TBIL, total bilirubin; DBIL, direct bilirubin; IBIL, indirect bilirubin; HDL, high density lipids; LDL, low-density lipoprotein; TC, total cholesterol; ApoA, apolipoprotein A; ApoB, apolipoprotein B.

**Table 4:** Predictive factors for MASLD: multivariable logistic regression.

Variable	P_Raw	P_FDR	OR (95%CI)
Male	<0.001	<0.001	7.76(4.07, 14.81)
BMI	<0.001	<0.001	1.42(1.30, 1.56)
*PNPLA3*-Ile148Met-GG	<0.001	<0.001	3.81(1.80, 8.07)
*PNPLA3*-Ile148Met-GC	0.704	0.704	1.13(0.59, 2.16)
*TM6SF2*-Glu167Lys-CT	0.002	0.003	3.62(1.57, 8.34)

Associations with FDR-corrected P-values (denoted as P_FDR) < 0.050 were considered statistically significant. All statistical tests were two-sided.

### 2.7 In silico analysis of protein stability

To assess the potential structural impact of the identified non-synonymous variants, we performed in silico analysis on the *PNPLA3* protein. The wild-type three-dimensional structure of human *PNPLA3* was retrieved from the AlphaFold Database (https://alphafold.ebi.ac.uk). The DynaMut2 web server (https://biosig.lab.uq.edu.au/dynamut2/) was employed to predict the changes in protein stability upon mutation (21). This tool calculates the change in folding free energy (ΔΔG, kcal/mol) between the wild-type and mutant structures, where a negative ΔΔG value indicates a destabilizing effect.

## 3 Results

### 3.1 Hardy–Weinberg equilibrium validation of genotype distributions

To ensure the reliability of subsequent genetic association analyses, we first validated the genotype distributions of all target SNPs for deviation from Hardy–Weinberg equilibrium (HWE) in both the MASLD cohort (n=196) and the control cohort (n=154). Detailed HWE test results for all variants are presented in **Supplementary Table 1**.

In the control cohort, no deviation from HWE was observed for any of the tested variants (all P > 0.050), indicating that the control group had a genetically balanced genotype distribution and good population representativeness.

In the MASLD cohort, the *PNPLA3*-Ile148Met variant showed a significant deviation from HWE (P < 0.001), while all other variants conformed to HWE (all P > 0.050). This deviation is likely biologically driven rather than technical, as the *PNPLA3* 148M (G) allele is a well-established and potent risk factor for steatotic liver disease. The strong enrichment of the high-risk GG genotype in our case group—a hallmark of genuine association—naturally leads to a departure from HWE expectations within the affected population (13, 14, 22). This observation, coupled with the absence of HWE deviation for this SNP in our control group and for all other SNPs in both groups, supports the integrity of our genotyping data and the specific, robust association of *PNPLA3*-Ile148Met with pediatric MASLD.

### 3.2 Differences in allele frequencies of 8 SNPs between the MASLD group and the control group

The allele frequencies of three SNP loci (*TM6SF2*-Glu167Lys, *PNPLA3*-Ile148Met, and *PNPLA3*-Lys434Glu) in children and adolescents with MASLD were significantly higher than those in the healthy control group (*TM6SF2*-Glu167Lys: P_FDR<0.001, OR = 3.04; *PNPLA3*-Ile148Met: P_FDR<0.001, OR = 2.50; *PNPLA3*-Lys434Glu: P_FDR=0.024, OR = 1.65; **Figure 1A**, **Table 1**), suggesting an association between these three SNPs and an increased risk of MASLD. In silico stability analysis further predicted that both *PNPLA3* missense variants (Ile148Met and Lys434Glu) have a destabilizing effect on the protein structure (ΔΔG = -0.55 and -0.23 kcal/mol, respectively).

In contrast, the variant allele frequencies of *GCKR*-Leu446Pro and *LEPR*-Lys109Arg were significantly lower in the MASLD group than in the control group (*GCKR*-p.Leu446Pro; T-C: P_FDR=0.009, OR = 0.64; *LEPR*-p.Lys109Arg; A-G: P_FDR=0.009, OR = 0.51). This implies that the variant sites of these two SNPs may serve as protective variants against MASLD, and individuals with the wild-type of these SNPs have a higher risk of developing MASLD.

### 3.3 Association between specific genotypes and MASLD susceptibility

Stratified analysis by genotype further confirmed the association of key variants with MASLD (**Figure 1B**, **Table 2**):

For *PNPLA3*-Ile148Met, the homozygous GG genotype and heterozygous GC genotype showed highly significant differences between the MASLD group and the control group (homozygous GG genotype: P_FDR<0.001, OR = 6.47; heterozygous GC genotype: P_FDR=0.007, OR = 0.43). This suggests that *PNPLA3*-Ile148Met exhibits a “recessive Mendelian-like inheritance pattern”—the homozygous GG genotype was significantly enriched in the MASLD group, and at the same time, it was observed that the heterozygous GC genotype had a significantly higher frequency in the control group. In addition, for *TM6SF2*-Glu167Lys, the CT genotype was significantly more frequent in the MASLD group than in the control group (P_FDR=0.003, OR = 2.80), a pattern similar to “dominant susceptibility”.

For other variants (e.g., *GCKR*-Leu446Pro, *LEPR*-Lys656Asn), although the ORs of their allele frequencies or genotypes deviated from 1 between the MASLD group and the control group, no statistically significant differences were observed in our sample (all P_FDR>0.050; **Table 2**). Notably, while the allele frequency of *PNPLA3*-Lys434Glu was significantly associated with MASLD risk (**Table 1**), its genotype frequencies did not reach statistical significance (**Table 2**), suggesting a potential dose-dependent effect that requires further validation.

### 3.4 Independent risk factors for pediatric MASLD

To identify independent risk factors for pediatric MASLD, a multivariate logistic regression model was constructed incorporating the pre-specified genetic models for *PNPLA3*-Ile148Met (recessive) and *TM6SF2*-Glu167Lys (dominant), with adjustment for potential confounding factors including age, sex, and body mass index (BMI). The final fitted model revealed that male sex, the *PNPLA3*-Ile148Met-GG genotype (under the recessive model), and the *TM6SF2*-Glu167Lys-CT genotype (under the dominant model, representing CT+TT carriers) were independent risk factors for MASLD (**Table 4**):

1. Male sex was associated with a 7.76-fold increased risk of MASLD (P_FDR<0.001);
2. Carriers of the *PNPLA3* rs738409 GG genotype had a 3.81-fold higher risk of developing MASLD compared with the combined GC/CC genotype group (P_FDR<0.001);
3. Carriers of the *TM6SF2*-Glu167Lys risk allele (CT/TT genotypes) had a 3.62-fold higher risk of MASLD compared to CC homozygotes (P_FDR=0.003).

Notably, after adjusting for confounding factors and under the recessive model, the *PNPLA3*-Ile148Met-GC genotype, when grouped with the CC genotype as the reference, showed no significant association with MASLD risk (P_FDR=0.704).

### 3.5 Differences in biochemical indicators among children with MASLD carrying different *PNPLA3* rs738409 genotypes

When comparing biochemical indicators between children with MASLD carrying different *PNPLA3* rs738409 genotypes (GG vs. GC), significant differences were observed in three key markers (**Table 3**): the levels of aspartate aminotransferase (AST), alanine aminotransferase (ALT), and total cholesterol (TC) were all significantly higher in the GG genotype group than in the GC genotype group (all P_FDR<0.001). No significant differences were found between the two genotypes in other indicators, such as body mass index (BMI), glucose, triglycerides (TG), and high-density lipoprotein (HDL) (all P_FDR>0.050).

Collectively, the results of this study confirm the following key findings: First, *PNPLA3*-Ile148Met (especially the GG genotype) and *TM6SF2*-Glu167Lys (especially the CT genotype) are significantly associated with an increased risk of MASLD in children, and both were identified as independent risk factors for pediatric MASLD after adjusting for confounding factors like age, sex, and BMI. Second, two SNPs were found to potentially act as protective variants against pediatric MASLD: *GCKR*-Leu446Pro and *LEPR*-Lys109Arg, as their allele frequencies were significantly lower in the MASLD group than in the control group (*GCKR*-Leu446Pro: P_FDR=0.009, OR = 0.64; *LEPR*-Lys109Arg: P_FDR=0.009, OR = 0.51), suggesting that carriers of these variant sites may have a reduced risk of MASLD.

## 4 Discussion

This case-control study provides robust evidence for the significant association between specific genetic variants and the risk of MASLD in Chinese children. Our principal findings confirm that *PNPLA3*-Ile148Met and *TM6SF2*-Glu167Lys are independent genetic risk factors in this population, thereby validating their central role in pediatric MASLD pathogenesis across ethnicities. Notably, we report the novel and paradoxical finding that the *GCKR-*Leu446Pro variant, widely recognized as a risk allele in Western adult cohorts, appears to confer a protective effect in our Chinese pediatric cohort. Furthermore, we identified a significant association of the *PNPLA3*-Lys434Glu variant with increased MASLD risk at the allele level, highlighting the potential contribution of additional variation within this pivotal gene. These results not only validate the roles of established genes but also underscore critical, population-specific characteristics in the genetic architecture of pediatric MASLD.

Our findings robustly reinforce the pivotal role of *PNPLA3* in MASLD susceptibility. The *PNPLA3*-Ile148Met variant (rs738409 G allele) was significantly enriched in our MASLD cohort and was identified as an independent risk factor, consistent with a vast body of international literature (22, 23). As previously reported (24–26), this variant reduces the TG hydrolase activity of *PNPLA3* while enhancing its lysophosphatidic acid acyltransferase (LPAAT) activity, thereby inhibiting lipid breakdown and promoting intrahepatic steatosis. Additionally, the significantly higher levels of aspartate aminotransferase (AST), alanine aminotransferase (ALT), and total cholesterol (TC) in children with the GG genotype further validate that this homozygous variant exacerbates liver injury—consistent with Stefano Romeo et al.’s observation that 148M allele carriers have elevated liver enzymes (27), and A. Kotronen et al.’s finding that rs738409 correlates with serum AST levels (28). This suggests *PNPLA3*-Ile148Met not only increases MASLD susceptibility but also associates with more severe hepatic dysfunction in pediatric patients. The observed deviation from Hardy-Weinberg equilibrium specifically in the MASLD case group for this variant is a recognized hallmark of a genuine, high-effect risk allele, as the over-representation of risk homozygotes disrupts genetic equilibrium in the affected population.

To further elucidate the potential structural and functional impacts of the identified *PNPLA3* variants, we performed in silico analysis. Using the DynaMut2 online tool, we compared the wild-type protein structure (obtained from the AlphaFold database, https://alphafold.ebi.ac.uk) with the mutant structures. The analysis predicted that both the Ile148Met and Lys434Glu substitutions are destabilizing to the *PNPLA3* protein structure. The Ile148Met variant showed a predicted stability change (ΔΔG) of -0.55 kcal/mol, while the Lys434Glu variant had a ΔΔG of -0.23 kcal/mol. A negative ΔΔG value indicates a decrease in folding stability, which may lead to protein misfolding, accelerated degradation, or a loss of proper enzymatic function (25, 29, 30). For the well-characterized Ile148Met variant, this computational prediction aligns with established experimental evidence showing that the mutation impairs triglyceride hydrolase activity and promotes aberrant lipid accumulation. Although the Lys434Glu variant has been less studied, its predicted destabilizing effect provides a plausible mechanistic basis for its association with MASLD risk observed at the allele level in our cohort (31). This suggests that, similar to the Ile148Met variant, the Lys434Glu substitution may compromise protein integrity and contribute to dysregulated hepatic lipid metabolism.

Similarly, our data confirm the importance of *TM6SF2*-Glu167Lys (rs58542926 T allele) as an independent risk factor for pediatric MASLD, supporting the conclusion of Goffredo et al (32). The dominant model of susceptibility observed in our cohort aligns with the proposed mechanism whereby the E167K variant causes misfolding and degradation of the *TM6SF2* protein (33), impairing very low-density lipoprotein (VLDL) assembly and triglyceride secretion from the liver. It is noteworthy that the frequency of the risk CT genotype in our Han Chinese MASLD group (24.49%) was substantially higher than that reported in some other ethnicities (e.g., 12.19% in Caucasians) (22), highlighting prominent population-specific genetic characteristics and underscoring the necessity of ethnic-specific genetic studies.

A pivotal and novel finding of our study is the protective association of the *GCKR*-Leu446Pro variant (OR = 0.64) in Chinese children with MASLD. This stands in stark contrast to its well-established role as a risk allele for hepatic steatosis in Western adult cohorts, highlighting the profound context-dependency of genetic risk. Mechanistically, the Leu446Pro variant causes a loss-of-function in the glucokinase regulator, leading to enhanced hepatic glycolysis and *de novo* lipogenesis (DNL)—the conventional explanation for its steatotic effect. We propose that in the specific context of childhood obesity—where dynamic growth and severe insulin resistance may not yet be fully entrenched—the resulting persistent glucokinase activity may paradoxically offer an advantage by improving hepatic glucose disposal. This potential benefit for systemic glucose homeostasis might temporarily outweigh the pro-steatotic DNL effect, yielding a net protective phenotype in our young cohort. This hypothesis is supported by functional evidence suggesting the variant’s pathogenicity is modulated by metabolic background, such as the presence of diabetes, which was rare in our population (34).

At the allele level, we also observed a significant association between the *PNPLA3*-Lys434Glu variant and increased MASLD risk. Although this variant did not exhibit significant genotype-level associations, its allele frequency enrichment in MASLD patients and its predicted destabilizing effect on protein structure (ΔΔG = -0.23 kcal/mol) suggest a potential role in disease susceptibility that warrants further investigation in larger cohorts.

From a clinical perspective, our identification of *PNPLA3*-Ile148Met and *TM6SF2*-Glu167Lys as independent risk factors, combined with the powerful non-genetic risk factor of male sex (OR = 7.76), provides a practical framework for refining early MASLD screening strategies in Chinese children. For instance, male children carrying the *PNPLA3* GG genotype or the *TM6SF2* CT/TT genotypes could be prioritized for more intensive lifestyle counseling and liver ultrasound monitoring, enabling timely intervention to prevent disease progression.

## 5 Limitations and future directions

While this study identifies significant genetic associations with pediatric MASLD, several limitations must be acknowledged. First, the cross-sectional nature of our design precludes the inference of causal relationships between the genetic variants and the development of MASLD. Second, the diagnosis of MASLD was based on ultrasonography rather than liver histology. Although ultrasound is a widely recommended, non-invasive first-line tool for population screening, it has limited sensitivity for detecting mild steatosis (<20% fat infiltration) and cannot assess liver fibrosis (35), a key determinant of disease progression. Third, our cohort was composed exclusively of Han Chinese children, which, while homogenous for initial discovery, limits the generalizability of our findings to other ethnic populations. Finally, we lacked detailed data on environmental and lifestyle factors, such as dietary habits (e.g., fructose intake) and physical activity levels, which are known to modulate genetic risk and are critical components of MASLD pathogenesis.

To address these limitations and build upon our findings, future research should prioritize several avenues. Prospective, longitudinal multiethnic cohorts are needed to establish the temporal sequence of genetic risk leading to MASLD and to validate the population-specific effects we observed, particularly for *GCKR*-Leu446Pro. The integration of multi-omics data—including genomics, gut microbiome profiling, and metabolomics—will be essential to elucidate the functional pathways through which these genetic variants operate and to uncover how they interact with environmental exposures. Ultimately, such integrated approaches are crucial for transitioning from genetic association to a mechanistic understanding of pediatric MASLD, paving the way for personalized risk prediction and targeted preventive strategies.

## 6 Conclusion

In summary, we show that the *PNPLA3*-Ile148Met and *TM6SF2*-Glu167Lys gene polymorphisms are independent risk factors that significantly increase the risk of MASLD in Chinese children. Additionally, the *PNPLA3*-Lys434Glu variant is associated with increased risk at the allele level. In contrast, *GCKR*-Leu446Pro and *LEPR*-Lys109Arg may confer protective effects. This study provides new evidence for genetic susceptibility to MASLD in the pediatric population.

## Data Availability

The datasets presented in this study can be found in online repositories. The names of the repository/repositories and accession number(s) can be found in the article/**Supplementary Material**.
